# Relationship Between Facial Parameters and Mandibular Third Molar Impactions: A Radiographic Cross-Sectional Study

**DOI:** 10.7759/cureus.98731

**Published:** 2025-12-08

**Authors:** Rohit Breh, Shitun Sarangi, Rama Shankar

**Affiliations:** 1 Orthodontics, Tata Main Hospital, Jamshedpur, IND; 2 Dentistry, Tata Main Hospital, Jamshedpur, IND; 3 Oral and Maxillofacial Surgery, Tata Main Hospital, Jamshedpur, IND

**Keywords:** cephalometrics, mandibular growth, orthodontics, third molar impaction, winter’s classification

## Abstract

Introduction: Mandibular third molar impaction is one of the most common dental anomalies with clinical implications ranging from pericoronitis to crowding. This study aimed to analyze the relationship between cephalometric parameters and third molar impaction types to predict eruption potential.

Methods: A cross-sectional radiographic analysis was performed on 244 patients (132 female patients, 112 male patients) at Tata Main Hospital, Jamshedpur. Orthopantomograms and lateral cephalograms were evaluated for Winter’s classification of impaction and cephalometric parameters including facial axis, mandibular plane angle, gonial angle, corpus length, and effective mandibular length. Statistical analysis included chi-square, Mann-Whitney U, and ANOVA tests. Results: Mesioangular impaction was predominant (68.2% in female patients, 73.2% in male patients). Significant associations were observed between Winter’s Class IV impactions and reduced mandibular corpus length (p=0.026) and increased gonial angle (p=0.044). Gender-specific patterns included Class I impaction linked to reduced corpus length in male patients (p=0.041) and low facial axis in female patients (p=0.048). Conclusion: Cephalometric parameters can provide valuable insight into third molar eruption potential. Routine lateral cephalograms may serve as predictive tools for identifying individuals at risk of impaction.

## Introduction

Mandibular third molar impaction is among the most frequently encountered dental anomalies and occurs when the tooth fails to attain its functional position within the dental arch due to spatial or developmental constraints. An impacted third molar is defined as a tooth that remains partially or completely embedded in bone or soft tissue for more than two years beyond its expected eruption time [1]. Insufficient retromolar space, aberrant eruption angulation, mandibular growth direction, and obstruction by adjacent second molars are the primary etiologic factors contributing to impaction [2,3]. The global prevalence of mandibular third molar impaction ranges between 18% and 40%, with notable variation across populations, and is influenced by craniofacial morphology, environmental factors, and genetic predisposition [4]. Clinically, impaction may predispose to pericoronitis, crowding, caries, periodontal defects, and adjacent second-molar resorption [5], highlighting the importance of early prediction of eruption pathways.

Craniofacial morphology has been shown to significantly influence the likelihood, angulation, and eruption direction of third molars. Prior studies have reported associations between mandibular length, gonial angle, mandibular plane inclination, and growth direction with impaction severity [6-8]. However, most investigations have evaluated isolated cephalometric parameters without examining integrated skeletal relationships. Furthermore, population-specific differences in craniofacial patterns warrant updated evaluation, particularly in South Asian cohorts, where evidence remains limited [9-11].

Winter’s classification continues to be one of the most widely adopted systems for characterizing mandibular third molar impactions based on eruption angulation and anticipated surgical difficulty [12]. Newer cephalometric approaches may enhance clinicians’ ability to anticipate eruption behavior and identify individuals at high risk for complex impactions [13]. Additionally, recent diagnostic research has emphasized the value of combining panoramic and lateral cephalometric findings to improve predictive accuracy for impacted third molars [14,15].

Aim

The aim of the study is to evaluate the relationship between cephalometric skeletal parameters and Winter’s classification of mandibular third molar impaction in orthodontic patients.

Objectives

The objectives of the study are to compare mandibular corpus length, gonial angle, mandibular plane angle, facial axis, and occlusal-plane inclination across different Winter’s impaction types; to assess gender-based variations in these cephalometric parameters; and to determine whether specific cephalometric patterns are associated with more severe impaction types.

Null hypothesis

There is no significant difference in cephalometric skeletal parameters across Winter’s mandibular third molar impaction types and no gender-based variation in these measurements.

## Materials and methods

**Figure 1:**
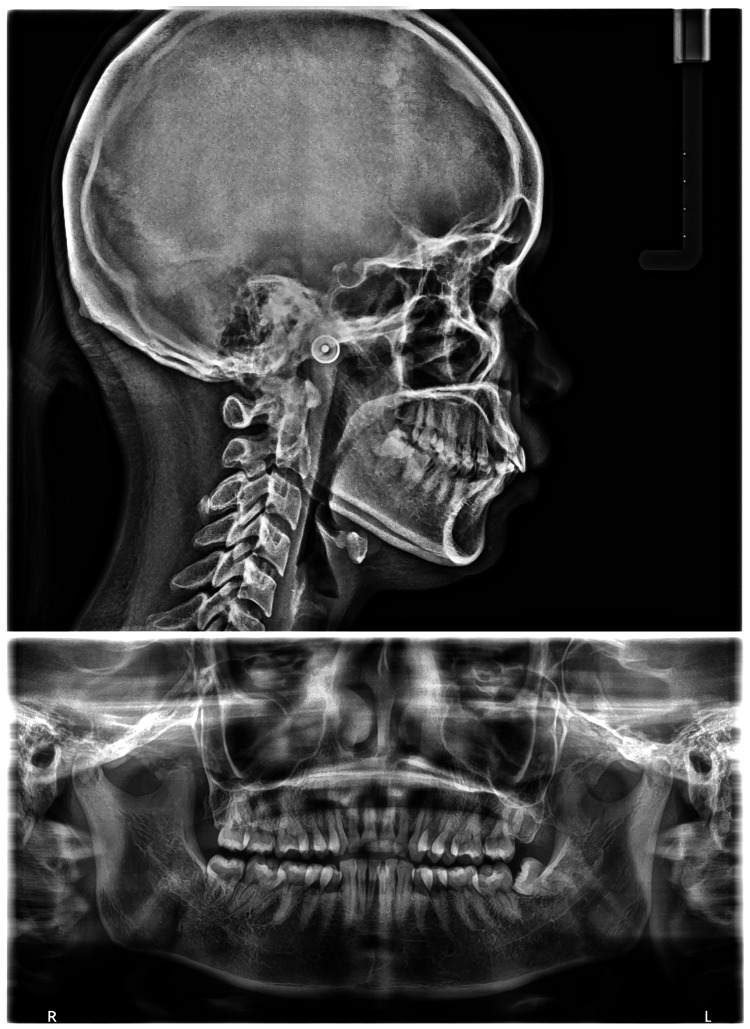
Representative Lateral Cephalogram and Panoramic Radiograph De-identified sample lateral cephalogram and panoramic radiograph demonstrating the imaging views used for cephalometric tracing and classification of mandibular third molar impaction. These images were taken in the department of Dentistry, Tata Main Hospital and contain no patient-identifiable information.

This radiographic cross-sectional study was conducted in the Department of Orthodontics at Tata Main Hospital, Jamshedpur. A total of 244 eligible pretreatment orthodontic patients who underwent routine lateral cephalometric and panoramic radiography between August 2020 and June 2024 were evaluated using consecutive sampling. Representative examples of the radiographs used in this study are shown in Figure 1.

Sample size estimation was based on previously reported cephalometric differences among mandibular third molar impaction types, with an effect size of 0.30, 80% power, and a 5% significance level, indicating a minimum requirement of 210 subjects [11,13]. To enhance statistical reliability and allow for potential data loss, the final sample consisted of 244 subjects.

Patients aged 16-25 years with complete permanent dentition, cervical vertebral maturation stage V or VI based on established criteria [16], and mandibular third molars exhibiting at least two-thirds root formation were included, following radiographic developmental standards [17,18]. Patients with craniofacial anomalies, systemic diseases affecting bone metabolism, developmental disorders, previous orthodontic treatment, significant maxillofacial trauma, or radiographs of poor diagnostic quality were excluded.

All radiographs were obtained using a standardized digital imaging unit with constant magnification. Lateral cephalograms were captured in natural head position with the Frankfort horizontal plane parallel to the floor. Panoramic radiographs followed routine hospital positioning protocols. All images were analyzed using Dolphin Imaging software (Dolphin Imaging & Management Solutions, Chatsworth, CA, USA) for analysis.

**Figure 2:**
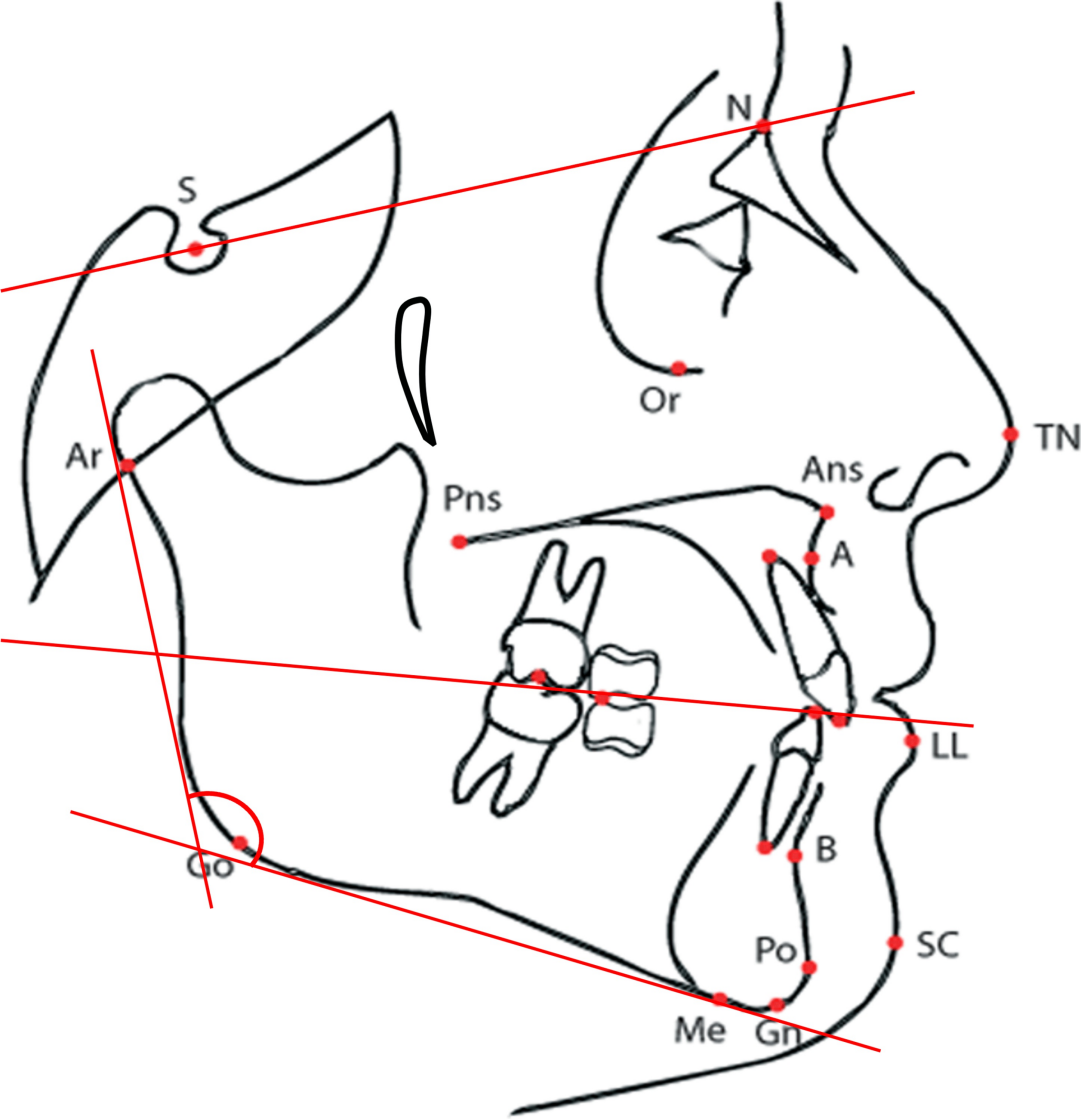
Cephalometric Landmarks Used for Measurement Cephalometric base outline for landmark identification. Image created by the authors.

Cephalometric measurements included mandibular corpus length, gonial angle, mandibular plane angle, facial axis, and occlusal-plane inclination. The cephalometric reference landmarks and planes used for these measurements are illustrated in Figure 2, and detailed definitions with corresponding landmarks/points are provided in Table 1.

**Table 1:** Definitions of Assessed Cephalometric Parameters and Their Anatomical Landmarks

Parameter	Definition	Landmarks/Points Used
Mandibular Corpus Length	Linear distance from Gonion to Gnathion	Go–Gn
Gonial Angle	Angle formed by ramus plane and mandibular plane	Ar–Go–Me
Mandibular Plane Angle	Angle between mandibular plane and SN plane	SN–GoMe
Facial Axis	Angle representing vertical skeletal pattern	Ba–N to Pt–Gn
Occlusal Plane Inclination	Inclination of occlusal plane relative to SN plane	SN–OP

**Figure 3:**
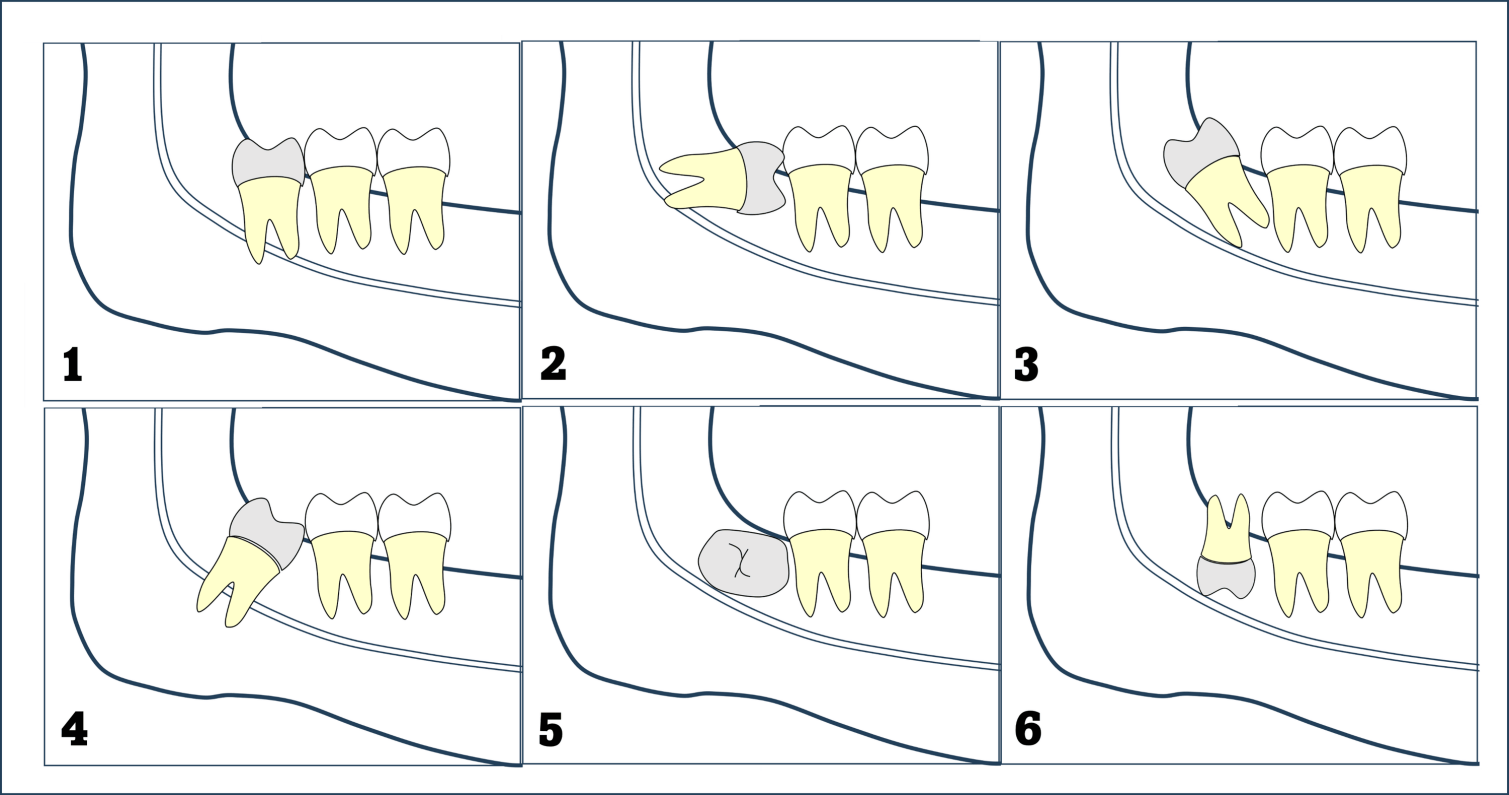
Winter’s Classification for Mandibular Third Molar Impaction Schematic representation of Winter’s classification of mandibular third molar impaction. Image created by the authors.

Mandibular third molar impaction was classified on panoramic radiographs according to Winter’s classification [12], based on the angular relationship of the mandibular third molar to the long axis of the second molar. A schematic representation of all six impaction types is presented in Figure 3.

All measurements were performed by a single calibrated examiner. To assess intra-observer reliability, 30 randomly selected radiographs were re-measured after two weeks. Intra-class correlation coefficients (ICC) ranged from 0.89 to 0.96 for linear variables and 0.86 to 0.94 for angular variables, confirming excellent reliability [12]. Random error was quantified using Dahlberg’s formula, yielding values between 0.7-1.1 mm for linear variables and 1.0°-1.4° for angular variables.

Normality of data distribution was tested using the Shapiro-Wilk test. Comparisons of cephalometric variables across Winter’s impaction types were performed using one-way ANOVA with Tukey’s post-hoc analysis. Gender-based comparisons were performed using independent-samples t-tests. Categorical distributions of impaction types were evaluated using chi-square tests. Statistical significance was set at p < 0.05.

Ethical approval for this study was obtained from the Institutional Ethics Committee of Tata Main Hospital, Jamshedpur (Reference No. TMH/2024/DENT/023; approval date: July 15, 2020). The requirement for informed consent was waived because anonymized radiographs were used.

## Results

Distribution of impaction types

The distribution of impaction types is presented in Table 2. A total of 244 subjects were evaluated, including 112 male patients (45.9%) and 132 female patients (54.1%). Mesioangular impaction was the most prevalent pattern, recorded in 172 subjects (70.5%), followed by distoangular impaction in 68 subjects (27.9%). Vertical impaction was identified in three subjects (1.2%), while only one subject (0.4%) exhibited horizontal impaction. The overall distribution of impaction type showed no meaningful difference between genders.

**Table 2:** Distribution of Mandibular Third Molar Impaction Types by Gender

Impaction Type	Female n (%)	Male n (%)	Total n (%)
Mesioangular	90 (68.2)	82 (73.2)	172 (70.5)
Distoangular	40 (30.3)	28 (25.0)	68 (27.9)
Vertical	2 (1.5)	1 (0.9)	3 (1.2)
Horizontal	0 (0.0)	1 (0.9)	1 (0.4)
Total	132 (100)	112 (100)	244 (100)

Cephalometric correlation with Winter’s classification

Cephalometric comparison across Winter’s impaction classes demonstrated clear skeletal distinctions. Class IV impactions were associated with the shortest mandibular corpus length and the steepest mandibular plane and gonial angles, reflecting a more vertical growth pattern. In contrast, subjects with Class I impaction exhibited a comparatively longer mandibular corpus length, a lower gonial angle, and a more horizontal growth tendency. These relationships were statistically significant, as presented in Table 3.

**Table 3:** Comparison of Cephalometric Parameters Across Winter’s Impaction Classes A one-way ANOVA test was used to compare mean cephalometric values across Winter’s impaction classes.

Winter’s Class	Mandibular Corpus Length (mm) Mean ± SD	Gonial Angle (°) Mean ± SD	Mandibular Plane Angle (°) Mean ± SD	Facial Axis (°) Mean ± SD	Test Statistic (F-value)	p-value
I	73.8 ± 3.1	122.4 ± 5.2	24.1 ± 3.6	90.6 ± 3.8	4.52	0.041
II	72.9 ± 2.8	124.3 ± 4.9	26.2 ± 3.9	89.5 ± 3.9	3.88	0.062
III	71.5 ± 3.5	126.1 ± 5.1	28.8 ± 4.2	88.3 ± 4.1	4.91	0.032
IV	70.2 ± 3.6	128.7 ± 4.7	30.5 ± 3.7	87.4 ± 3.6	5.77	0.026

Gender-based cephalometric interpretation

Gender-specific analysis revealed selective associations within Winter’s classes. Among male patients, those with Class IV impaction demonstrated a significantly steeper mandibular plane angle compared with female patients in the same category (p = 0.001). Among female patients, Class I impaction was associated with a significantly reduced facial axis angle relative to male patients (p = 0.048). No significant sexual dimorphism was observed in gonial angle or mandibular corpus length in any impaction class. Gender-based comparisons of significant cephalometric parameters are presented in Table 4.

**Table 4:** Gender-Based Comparison of Significant Cephalometric Parameters Statistical test used: Independent-samples t-test Significance level: p < 0.05 *Indicates a statistically significant difference. Gender-based comparison of significant cephalometric parameters within each Winter’s class. An independent-samples t-test was used for statistical analysis.

Cephalometric Variable	Male (Mean ± SD)	Female (Mean ± SD)	t-value	p-value
Mandibular Plane Angle	34.8 ± 4.1	31.2 ± 3.8	3.39	0.001*
Facial Axis Angle	88.4 ± 3.2	86.1 ± 3.4	2.01	0.048*
Gonial Angle	122.6 ± 6.0	123.1 ± 5.8	0.78	0.43 (NS)
Mandibular Corpus Length (mm)	72.4 ± 4.5	72.9 ± 4.3	0.92	0.36 (NS)

## Discussion

The present study examined the association between mandibular third molar impaction patterns and underlying skeletal morphology, revealing significant cephalometric distinctions across Winter’s classification categories. Mesioangular impaction emerged as the most prevalent type, consistent with well-established global and regional literature indicating that forward mandibular growth and limited retromolar space commonly predispose to mesial angulation of the third molar [2,3]. The distribution pattern observed in the present sample parallels findings reported in diverse populations, suggesting that the skeletal and dental determinants of impaction are broadly consistent across ethnic groups.

A key finding of this study was the strong association between deeper impaction categories-particularly Class IV-and cephalometric features characteristic of vertical or hyperdivergent facial patterns. Subjects with severe impactions exhibited steeper mandibular plane angles, increased gonial angles, and reduced mandibular corpus length, all of which decrease available posterior arch length and limit eruption potential. These skeletal constraints align with classic and contemporary work highlighting the importance of mandibular rotation, posterior space availability, and growth direction in predicting third molar eruption [6-11]. In contrast, individuals with Class I impaction showed comparatively favorable horizontal growth tendencies, including increased corpus length and reduced mandibular plane inclination, supporting the notion that sagittal and vertical skeletal development jointly influence eruption pathways.

Gender-based differences were modest but clinically relevant. Male patients demonstrated significantly steeper mandibular plane angles within deeper impaction groups, whereas female patients with Class I impactions exhibited lower facial axis angles. These observations are consistent with studies reporting subtle cephalometric sexual dimorphism, particularly in angular measurements, without major differences in overall impaction susceptibility [13,14]. Such variations likely reflect normal developmental differences rather than true sex-based predispositions.

Collectively, the findings of this study reinforce that mandibular third molar impaction is multifactorial, with skeletal morphology exerting a substantial predictive influence. The clear cephalometric distinctions across Winter’s impaction classes confirm the diagnostic utility of lateral cephalograms in identifying individuals at increased risk of deeper or more complex impaction. Recent literature emphasizes the growing relevance of cephalometric parameters in predictive modeling for eruption potential [13-15], and the present results further support the incorporation of skeletal indicators into early orthodontic assessment and treatment planning.

Clinical implications

The results of this study underscore the importance of integrating cephalometric evaluation into routine orthodontic screening for predicting mandibular third molar eruption. Parameters such as increased mandibular plane angle, larger gonial angle, and reduced mandibular corpus length may help clinicians identify patients at higher risk of severe or deep impaction. Early recognition of these skeletal predictors enables targeted monitoring, space management strategies, or the consideration of prophylactic extraction when appropriate. Incorporating cephalometric indicators into pre-treatment evaluation enhances diagnostic accuracy, supports individualized treatment planning, and may reduce the likelihood of complications associated with impacted third molars. This approach aligns with contemporary orthodontic protocols emphasizing preventive assessment and growth-based prediction to optimize patient outcomes.

Study limitations

This study has several limitations. As a single-center investigation, the findings may not fully represent broader population variability. The reliance on two-dimensional radiography may limit precision when compared with three-dimensional imaging modalities such as cone-beam computed tomography. Although intra-observer reliability was assessed and confirmed to be strong, the absence of inter-observer reliability testing may reduce reproducibility. Additionally, the consecutive sampling method may introduce selection bias. Future multicenter, longitudinal studies using advanced imaging techniques are recommended to validate cephalometric predictors of impaction and further refine eruption prediction models.

## Conclusions

The present study demonstrates that mandibular third molar impaction is closely related to underlying skeletal morphology, with clear cephalometric distinctions across Winter’s classification categories. Deeper impactions, particularly Class IV, were associated with a vertical growth pattern characterized by steeper mandibular plane angles, increased gonial angles, and reduced mandibular corpus length, all of which reduce posterior arch space and limit eruption potential. In contrast, individuals with Class I impactions exhibited more favorable horizontal growth tendencies, reflecting greater corpus length and reduced mandibular plane inclination. Gender-based differences were minimal and limited to selected cephalometric variables.

Overall, these findings reinforce the predictive value of cephalometric parameters, especially mandibular plane angle, gonial angle, and corpus length, in assessing the likelihood of mandibular third molar eruption. Incorporating these skeletal indicators into orthodontic evaluation may improve early identification of patients at increased risk of severe or deep impaction and support more informed clinical decision-making.
